# Ginsenoside Rd attenuates breast cancer metastasis implicating derepressing microRNA-18a-regulated Smad2 expression

**DOI:** 10.1038/srep33709

**Published:** 2016-09-19

**Authors:** Peiwei Wang, Xiaoye Du, Minqi Xiong, Jingang Cui, Qinbo Yang, Wenjian Wang, Yu Chen, Teng Zhang

**Affiliations:** 1 Yueyang Hospital & Clinical Research Institute of Integrative Medicine, Shanghai University of Traditional Chinese Medicine, Shanghai 200437, China

## Abstract

Metastasis remains a major cause of mortality and poor prognosis in breast cancer patients. Anti-metastatic therapies are in great need to achieve optimal clinical outcome in breast cancer patients. Panax Notoginseng Saponins (PNS) has previously been shown to inhibit breast cancer metastasis in mouse. Here the potential anti-metastatic effect of one of the chemical compounds of PNS, ginsenoside Rd (Rd), was further evaluated in mouse mammary carcinoma 4T1 cells. The results revealed that Rd treatment dose-dependently suppressed cell migration and invasion in cultured 4T1 cells. In 4T1 cell-inoculated mice, Rd treatment led to decreased number of tumor lesions in lungs in both spontaneous and experimental metastasis models. Rd treatment resulted in increased expression of Smad2 in cultured 4T1 cells and in tumors grown from inoculated 4T1 cells. Rd treatment decreased the expression of microRNA (miR)-18a in cultured 4T1 cells and in tumors derived from inoculated 4T1 cells. Smad2 was further verified to be a direct target of miR-18a in 4T1 cells. The significant impact of Rd on counteracting miR-18a-medidated downregulation of Smad2 expression was also demonstrated. Together, the current work shows for the first time that Rd treatment attenuates breast cancer metastasis in part through derepressing miR-18a-mediated Smad2 expression regulation.

Breast cancer is the leading type of cancer in women worldwide. Advances in cancer treatment including surgery, chemotherapy, radiotherapy and biotherapy have increased the survival rate in cancer patients including those inflicted with breast cancer. However, metastasis remains an obstacle for optimal clinical management to further reduce the mortality rate and improve prognosis in breast cancer patients. Thus active efforts are still required to develop therapeutics to limit the metastasis in breast cancer patients.

Both clinical findings and experimental evidence have demonstrated that transforming growth factor (TGF) β signaling plays important roles in tumorigenesis and metastasis of breast cancer, either being oncogenic or tumor suppressive^1^,^2^,^3^. Typically, pathophysiological effects of TGFβ are executed by transcription factors known as Smads^4^. After binding of TGFβ to its heterodimeric receptor TGFβ type 2 receptor (TGFβR2), TGFβ type 1 receptor (TGFβR1) is transactivated. Activated TGFβR1 phosphorylates Smad2 and Smad3, which subsequently associate with Smad4, translocate to the nucleus, bind to the “CAGA” consensus sequence and regulate the transcription of target genes. TGFβ signaling pathway is a promising target in cancer therapy. Indeed, several compounds modulating this signaling pathway are under preclinical development or being evaluated in clinical trials^5^.

microRNA (miRNA)s are endogenous, single-strand non-coding RNAs with approximate length of 22 nucleotides. miRNAs play important roles in regulating gene expression mainly by targeting 3′-untranslated region (3′-UTR) of RNA transcripts, resulting in mRNA degradation or translational repression^6^. The functional significance of miRNA-mediated gene expression is supported by its implication in diverse pathophysiological processes^7^. miRNA-mediated regulation of TGFβ/Smad signaling has recently been demonstrated^8^. TGFβ superfamily receptors^9^,^10^, Smads^11^,^12^,^13^ and multiple components of the TGFβ signaling pathway have been shown to be regulated by miRNAs. For instance, Smad2 has been revealed to be a direct target of miR-18a in neuroblastoma cells. miR-18a is a member of the miR-17-92 cluster that is noted for its oncogenic potentials. miR-18a is implicated in the progression of various cancers including breast cancer^14^,^15^, colorectal cancer^16^, pancreatic cancer^17^, prostate cancer^18^ and nasopharyngeal cancer^19^.

Panax Notoginseng has been extensively used in China as a therapeutic agent to treat a wide range of diseases including cancer^20^. Our previous studies have shown that Panax Notoginseng Saponins (PNS), the major class of chemical component of the whole Panax Notoginseng extract, inhibits breast cancer metastasis in mouse^21^. We have also demonstrated that PNS treatment suppresses the tumor growth and decreases miR-18a expression in tumors derived from Lewis lung carcinoma cells^22^. The batch of PNS used by our previous studies mainly consists of ginsenoside Rb1, Rg1, Rd, Rh1 and notoginsenoside R1. However, which chemical component of PNS is pharmacologically active in suppressing breast cancer metastasis and the possible implication of miR-18a-mediated Smad2 expression regulation in this process remains to be investigated.

Ginsenoside Rd (Rd) has mainly been revealed to be neuroprotective and cardioprotective^23^,^24^,^25^. Rd has been shown to inhibit hepatocellular carcinoma HepG2 cell metastasis^26^ and gastric and breast cancer cell proliferation and survival *in vitro*^27^. In the current study, we set out to examine the potential activity of Rd in breast cancer metastasis by examining mammary carcinoma 4T1 cell migration and invasion *in vitro* and 4T1 cell metastasis *in vivo*. The results revealed significant anti-metastatic effects of Rd both in cultured 4T1 cells and in tumors derived from 4T1 cells. Rd treatment also decreased miR-18a expression and increased the level of Smad2 in cultured 4T1 cells and in 4T1-derived tumors. Furthermore, Rd treatment specifically increased the luciferase reporter activity of Smad2 3′-UTR containing miR-18a seed region, supporting the implication of miR-18a-mediated Smad2 regulation in the anti-metastatic effect of Rd in 4T1 mammary carcinoma cells.

## Results

### Rd treatment attenuated the growth of cultured 4T1 cells

**Figure 1:**
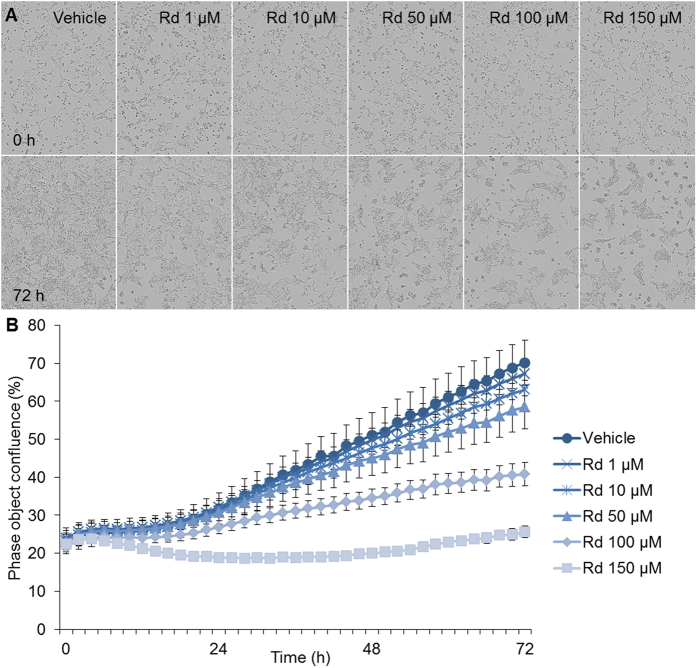
Rd inhibited the viability of 4T1 cells in a dose- and time-dependent manner. 4T1 cells were seeded in a 96-well plate in the number of 10,000 cells per well in the presence of vehicle or Rd at indicated concentrations including 1, 10, 50, 100 and 150 μM, respectively. Microphotographs (**A**) were recorded and percentage confluence (**B**) was monitored at 1 h interval using the high definition automated imaging system from IncuCyte (Essen BioScience). The experiments were performed in triplicate.

The effect of Rd treatment on the growth of cultured 4T1 cell was first examined by a non-labeling cell viability assay. Cultured 4T1 cells were incubated for 72 h in the presence of vehicle or Rd at concentrations ranging from 1 to 150 μM. As shown in Fig. 1, no overt decrease of cell number was observed when the cells were treated by Rd at 1 and 10 μM, respectively. However, compared to that from the vehicle control, decrease in 4T1 cell number became evident at 24 h when Rd was added at 100 and 150 μM, respectively. By 72 h, treatment with Rd at 50, 100 and 150 μM resulted in significant decrease in 4T1 cell number in a dose-dependent manner. Impairment in cell viability was also observed in human breast cancer MDA-MB-231 cells in the presence of Rd treatment (see Supplementary Fig. S1).

### Rd treatment inhibited 4T1 cell migration

**Figure 2:**
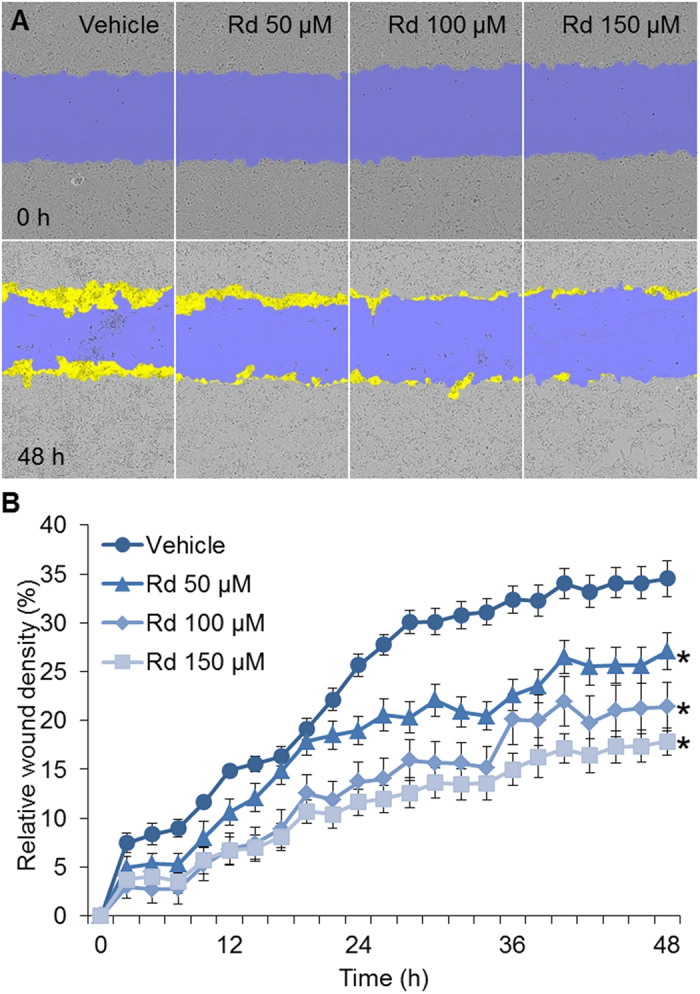
Rd inhibited the migration of 4T1 cells. 4T1 cells were seeded in triplicate into Essen ImageLock 96-well plates in the number of 20,000 cells per well. Cells were then serum-starved for 6 h and confluent cell layers were scratched using Essen Wound Maker to generate wounds approximately 600 μm wide, which was followed by treatment with vehicle or Rd at indicated concentrations including 50, 100 and 150 μM, respectively. Images were automatically acquired and registered by the IncuCyte ZOOM imaging system using time-lapse bright field microscopy. Typical kinetic updates were recorded at 2 h intervals for 48 h. (**A**) Representative images of wound mask of 4T1 cells treated with Rd in scratch/wound migration assay. The initial wound mask (in blue) and wound closure (in yellow) were recorded by IncuCyte. (**B**) RWD was generated to quantify 4T1 cell migration. *Compared to vehicle control, *p* < 0.05.

Next, the effect of Rd treatment on 4T1 cell migration dynamics was examined. As shown in Fig. 2, a significant decrease in 4T1 cell migration was observed when cells were treated with Rd at the dose as low as 50 μM, with 150 μM of Rd treatment displaying the most significant effect. Moreover, when Rd was applied at the concentration of 50 μM, the inhibitory effect of Rd treatment on 4T1 cell migration was noted as early as 24 h post initiation of treatment when no overt suppression of cell growth was observed (Fig. 1B), suggesting an effect of Rd treatment on 4T1 cell migration independent of its effect on growth inhibition. At 48 h, significant reduction in wound closure was observed in Rd-treated cells. As shown in Fig. 2B, compared to relative wound density (RWD) of 35% observed from vehicle-treated cells, RWD of 27%, 21% and 17% were observed when cells were treated with Rd at the dose of 50 μM, 100 μM and 150 μM, respectively. Inhibition of migration was also observed in human breast cancer MDA-MB-231 cells when Rd treatment was applied (see Supplementary Fig. S2). These results indicated a significant effect of Rd treatment on breast cancer cell migration *in vitro*.

### Rd treatment attenuated the metastatic potential of 4T1 cell *in vitro*

**Figure 3:**
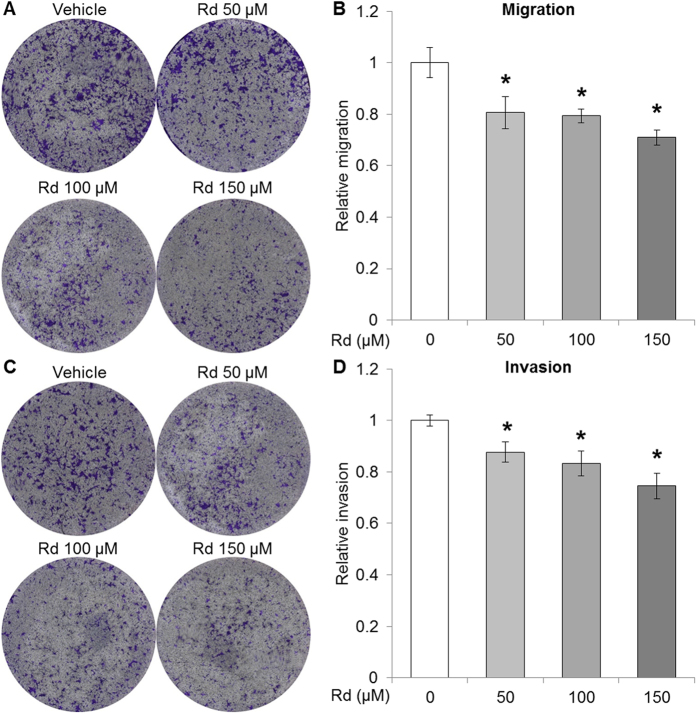
Rd suppressed the metastatic potential of 4T1 cells. 4T1 cells were resuspended in serum-free RPMI-1640 medium containing vehicle or Rd at indicated concentrations, which was followed by seeding in the top chamber of the transwell with or without matrigel coating to assess the migration and invasion of 4T1 cells, respectively. RPMI-1640 supplemented with 10% FBS was added to the lower chamber. After incubation at 37 °C for 24 h, cell viability was first determined by the CellTiter 96 AQueous Non-Radioactive Cell Proliferation MTS assay. Cells migrated through the filters were quantified by measuring absorbance at 540 nm by a microplate reader after crystal violet staining. Cell migration was assessed by normalizing against cell viability. For evaluation of cell invasion, measurement from invasion assay was normalized against that from migration assay. (**A**) Representative merged whole well image of each insert captured in transwell migration assay. (**B**) Relative migration from indicated treatment was presented by comparing migration from each experimental condition against that of vehicle-treated 4T1 cells. (**C**) Representative merged whole well image of each insert captured in transwell invasion assay. (**D**) Relative invasion from indicated treatment was presented after comparing invasion from each experimental condition against that of vehicle-treated 4T1 cells. *Compared to that from vehicle control, *p* < 0.05.

To better understand the impact of Rd treatment on breast cancer cell metastatic potential, transwell assay was further performed in the absence and presence of matrigel coating for the assessment of migration and invasion, respectively. To clarify the influence of impaired cell viability on metastatic potential, cell viability assessment was included in parallel. As shown in Fig. 3A,B, compared to that from the vehicle control, Rd treatment resulted in attenuated migration at 50, 100 and 150 μM, respectively. Moreover, the invasion of 4T1 cells was significantly alleviated by Rd treatment delivered at 50, 100 and 150 μM, respectively (Fig. 3C,D). To further validate the impact of Rd on the metastatic potential of 4T1 cells, the expression of genes regulating tumor metastasis was also examined. Significantly increased expression of anti-metastatic tissue inhibitor of metalloproteinase-2 (Timp2)^28^ was observed in 4T1 cells treated with Rd at 50, 100 and 150 μM, respectively (see Supplementary Fig. S3A). A significant decrease in the expression of pro-metastatic matrix metalloproteinase-3 (MMP3)^29^ was observed in 4T1 cells treated with Rd at 150 μM (see Supplementary Fig. S3B). These results collectively supported the suppressive effect of Rd treatment on the metastatic potential of 4T1 cells *in vitro*.

### Rd treatment suppressed 4T1 cell lung metastasis *in vivo*

**Figure 4:**
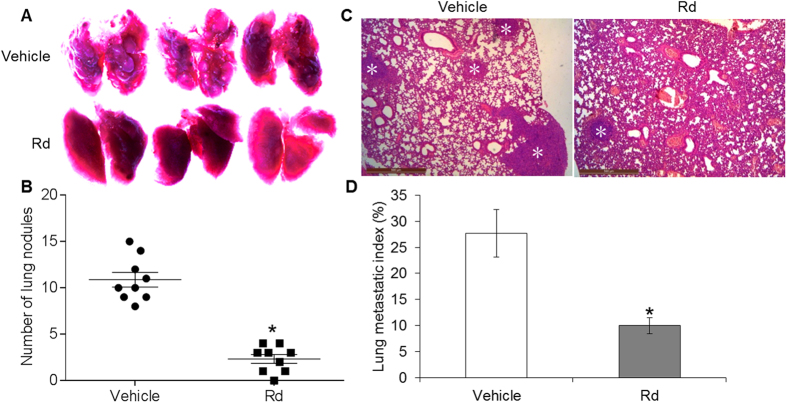
Rd inhibited the lung metastasis of inoculated 4T1 cells in spontaneous metastasis model. 4T1 cells were inoculated in the number of 1 × 10^6^ into the inguinal mammary fat pad of each BALB/c mouse. Mice were then treated daily via intraperitoneal injection with either Rd at the dose of 50 mg/kg bw or 0.9% saline vehicle. The mice were euthanized 3 weeks later. The number of lung colonies was recorded under light microscope (magnification 50×) (**A**) and the counted number of lung nodules were adjusted by tumor burden (**B**). (**C**) Tissue sections were examined by H&E staining and observed under light microscope. (**D**) Microscopic lung metastatic lesions were quantified by counting the total tissue area per lung section (D1) and micro-metastasis present in the same area (D2) using Image-Pro Plus software. The metastatic index was defined as the ratio of D2/D1. Scale bar: 500 μm. Asterisks indicate metastatic lesions. *Compared to that from the vehicle control, *p* < 0.05.

Inoculated 4T1 cells have the capacity to grow into solid tumor with spontaneous lung metastasis. We thus continued to evaluate the effect of Rd treatment on spontaneous lung metastasis in the mice inoculated with 4T1 cells. The mice were treated with either vehicle of 0.9% saline or Rd at the dose of 50 mg/kg bw. The dose of Rd for mouse treatment was selected based on the content of Rd in PNS and the effective dose of PNS on suppressing the lung metastasis in this model as previously described. As shown in Fig. 4A, compared to that from the vehicle-treated mice, significantly decreased number of tumor nodules was observed in the lungs of Rd-treated mice. Histological examination was performed to visualize the microscopic tumor lesions in the lungs from both Rd- and vehicle-treated mice. As shown in Fig. 4B, significantly reduced lung metastatic index was observed in Rd-treated mice compared to that from vehicle-treated mice. However, when the primary tumor growth was assessed, decreased tumor volume and tumor weight were noted in Rd-treated mice compared to that from the vehicle-treated mice (see Supplementary Fig. S4). The average tumor weight from vehicle-treated and Rd-treated mice was 0.61 g and 0.45 g, respectively, and an inhibition rate of 26.23% was thus observed as a result of Rd treatment. These results suggest the possibility that decreased lung metastasis may in part result from the inhibitory effect of Rd on primary tumor growth.

To clarify the effect of Rd treatment on lung metastasis *in vivo*, experimental metastasis model was further adopted. The survival rate of vehicle-treated and Rd-treated mice was 62.5% and 100%, respectively, indicating that mouse survival was improved by Rd treatment (see Supplementary Fig. S5).

**Figure 5:**
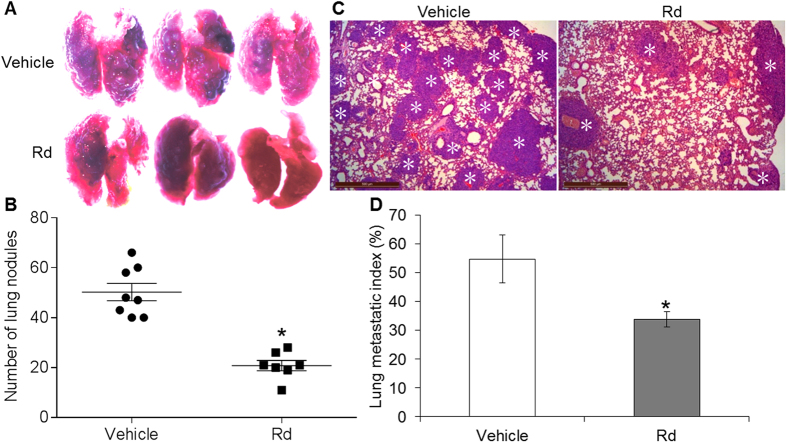
Rd inhibited the lung metastasis of inoculated 4T1 cells in experimental metastasis model. 4T1 cells were delivered in the number of 5 × 10^5^ to BALB/c mice through lateral tail vein injection. Mice were then treated daily with either Rd at the dose of 50 mg/kg bw or 0.9% saline vehicle via intraperitoneal injection. The mice were euthanized 2 weeks later. Lung metastasis nodules were photographed (**A**) and further quantified (**B**) under light microscope (magnification 50×). (**C**) Microscopic metastatic lesions in lungs were revealed after H&E staining of lung tissue sections. (**D**) Microscopic lung metastatic lesions were quantified by counting the total tissue area per lung section (D1) and micro-metastasis present in the same area (D2) using Image-Pro Plus software. The metastatic index was defined as the ratio of D2/D1. Scale bar: 500 μm. Asterisks indicate metastatic lesions. *Compared to that from the vehicle control, *p* < 0.05.

Moreover, macroscopic and microscopic examinations revealed significantly reduced number of lung metastatic nodules (Fig. 5A) and metastatic lesions (Fig. 5B) in Rd-treated mice compared to that from the vehicle-treated mice. These results suggested that independent of its effect on inhibiting primary tumor growth, Rd treatment attenuates lung metastasis of 4T1 cells *in vivo*.

### Rd treatment decreased miR-18a expression in cultured 4T1 cells and 4T1 cell-derived tumors

**Figure 6:**
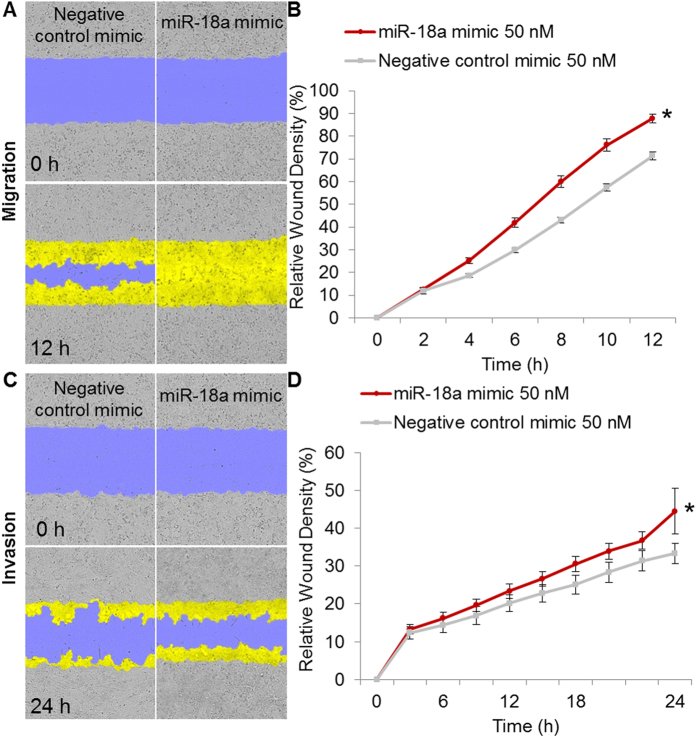
miR-18a promoted 4T1 cell migration and invasion. 4T1 cells were seeded in Essen ImageLock 96-well plates at 2 × 10^4^ cells per well. Cells were transfected with negative control mimic or miR-18a mimic at concentration of 50 nM for 24 h. The monolayer of confluent cells was then scratched using the Essen WoundMaker to generate wound approximately 600 μm wide. Wounds were subject to evaluation of cell migration, which was monitored for 12 h at 2 h intervals (**A**,**B**). Or 50 μl of serum-free RPMI 1640-diluted matrigel was coated on the scratched wound for further evaluation of cell invasion, which was monitored for 24 h at 2 h intervals (**C,D**). Wound images were automatically acquired and registered by the IncuCyte ZOOM imaging system using time-lapse bright field microscopy. The initial wound mask (blue) and wound closure (yellow) were recorded and measured using IncuCyte. (**A**) Representative images of wound mask of 4T1 cells transfected with negative control mimic or miR-18a mimic in the migration assay. (**B**) RDW matrices of 4T1 cells transfected with negative control mimic or miR-18a mimic in migration assay. (**C**) Representative images of wound mask of 4T1 cells transfected with negative control mimic or miR-18a mimic in invasion assay. (**D**) RDW matrices of 4T1 cells transfected with negative control mimic or miR-18a mimic in invasion assay. *Compared to that from negative control mimic-transfected cells, *p* < 0.05.

**Figure 7:**
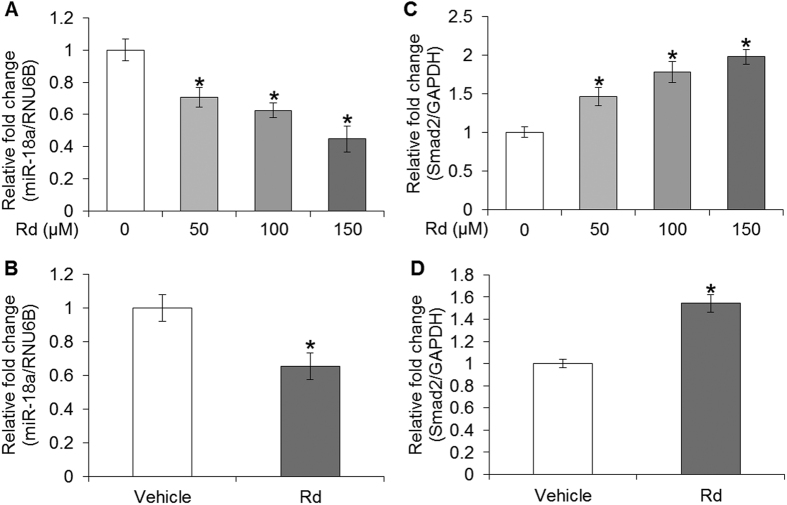
Rd reduced the expression of miR-18a and increased the expression of Smad2 in cultured 4T1 cells and in 4T1 cell-derived tumors in mice. miR-18a expression in the absence or presence of Rd treatment was analyzed using real-time PCR in cultured 4T1 cells (**A**) and 4T1 cell-derived tumors (**B**). Real-time PCR was also performed to analyze the relative mRNA expression of Smad2 in vehicle or Rd-treated 4T1 cells (**C**) and in 4T1-derived tumors after vehicle or Rd treatment (**D**). Relative fold change in the expression level was plotted against that of vehicle control. *Compared to that from the vehicle control, *p* < 0.05.

To elucidate whether miR-18a was involved in Rd-mediated attenuation of breast cancer metastasis, the impact of miR-18a expression on 4T1 cell metastatic potential was first examined. The data showed that at a dose of miR-18a mimic transfection that resulted in no significant impairment of 4T1 cell survival (see Supplementary Fig. S6), the migration (Fig. 6A,B) and invasion (Fig. 6C,D) of 4T1 cells were significantly promoted compared to that from the cells transfected with negative control mimic. In addition, miR-18a expression in response to Rd treatment was analyzed. As shown in Fig. 7A, Rd treatment dose-dependently caused significant decrease in the expression of miR-18a in cultured 4T1 cells. Similarly, reduced level of miR-18a was observed in 4T1 cell-derived tumors from Rd-treated mice compared to that from vehicle-treated mice (Fig. 7B). These results indicated that Rd-treatment leads to decreased miR-18a expression both *in vitro* and *in vivo*. It is worth noting that the expression of members of miR-17-92 cluster encoded by the MIR17HG gene is under the direct regulation of c-MYC, an important transcriptional factor implicated in cell proliferation, growth and apoptosis. Meanwhile, cell cycle regulator E2F1 is not only regulated by c-MYC, but also negatively regulated by two members of miR-17-72 cluster, namely miR-17-5p and miR-20a^30^. To clarify the possibility that cell cycle arrest might contribute to downregulated expression of miR-18a in Rd-treated 4T1 cells, the cell cycle distribution was analyzed in 4T1 cells treated with vehicle or Rd at 10, 50, 100 and 150 μM, respectively. The results revealed a decrease by about 10% and 14% in the proportion of cells at G0/G1 phase as a result of Rd treatment delivered at 100 and 150 μM, respectively. Meanwhile, the proportion of S-phase cells was increased by about 7% and 9% by Rd treatment given at 100 and 150 μM, respectively. However, no significant changes in cell cycle distribution were observed when 4T1 cells were exposed to Rd at 50 μM (see Supplementary Fig. S7). Given that significant reduction of miR-18a expression was observed in 4T1 cells treated by Rd at 50 μM (Fig. 7), it is possible that decreased expression miR-18a in Rd-treated 4T1 cells was independent of the influence of Rd on cell cycle distribution. However, when Rd was delivered at higher doses, changes in cell cycle distribution might exert additionally impact on the expression of miR-18a in 4T1 cells.

### Rd treatment altered the level of Smad2 in cultured 4T1 cells and 4T1 cell-derived tumors

**Figure 8:**
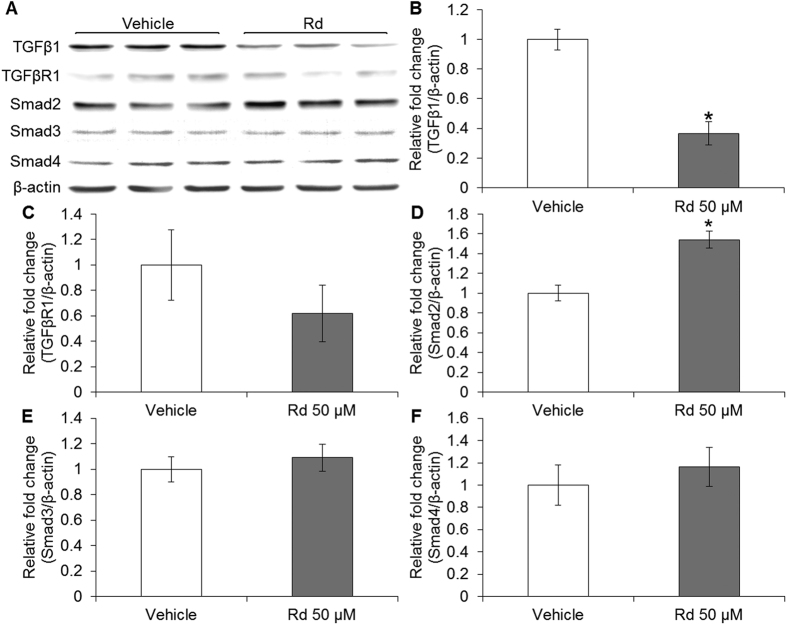
Rd treatment altered the levels of Smad2 and TGFβ1 in cultured 4T1 cells. (**A**) Protein expression of TGFβ1, TGFβR1 and Smads in Rd-treated 4T1 cells was analyzed by Western blot. Signals were visualized and recorded by UVP BioSpectrum imaging system. The quantification of the protein levels of TGFβ1 (**B**), TGFβR1 (**C**), Smad2 (**D**), Smad3 (**E**) and Smad4 (**F**) was performed by Launch VisionWorks LS. β-actin was used as an internal control for normalization. Relative fold change in the expression level was plotted against that of vehicle control. *Compared to that from the vehicle control, *p* < 0.05.

**Figure 9:**
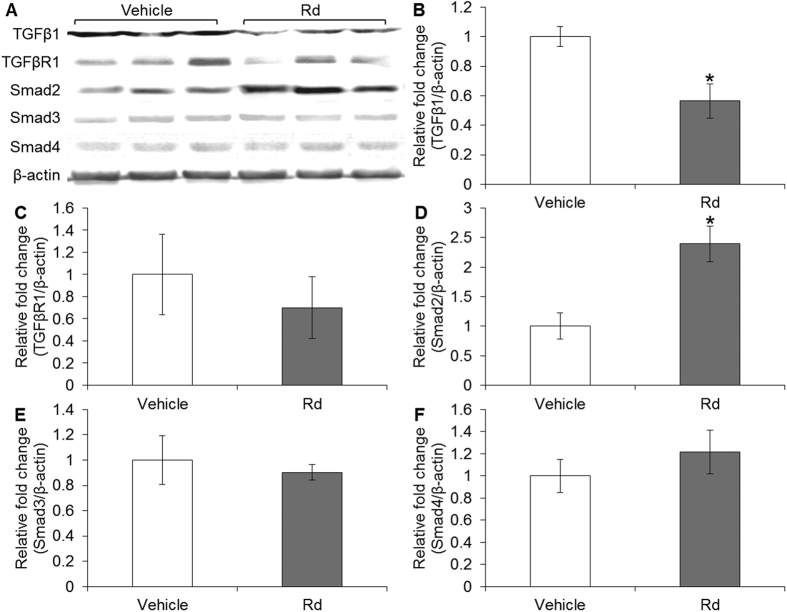
Rd treatment altered the levels of Smad2 and TGFβ1 in 4T1 cell-derived tumors. Total proteins from homogenized 4T1 cell-derived tumors were subjected to Western blot analyses to examine the protein levels of TGFβ1, TGFβR1 and Smads after vehicle or Rd treatment in BALB/c mice. Signals were visualized and recorded by UVP BioSpectrum imaging system (**A**). The quantification of the protein levels of TGFβ1 (**B**), TGFβR1 (**C**), Smad2 (**D**), Smad3 (**E**) and Smad4 (**F**) was performed by Launch VisionWorks LS. β-actin was used as an internal control for normalization. Relative fold change in the expression level was plotted against that of vehicle control. *Compared to that from the vehicle control, *p* < 0.05.

Given that TGF-β/Smad signaling is implicated in breast cancer metastasis^31^ and miR-18a has been demonstrated to directly regulate Smad2 in neuroblastoma cells^28^, we hypothesized that miR-18a-mediated regulation of Smad2 expression is involved in the suppressive effect of Rd treatment on breast cancer metastasis. To test this hypothesis, the expression of Smad2 in 4T1 cells and 4T1 cell-derived tumors was first examined in the presence or absence of Rd treatment. As shown in Fig. 7C, compared to that from vehicle-treated 4T1 cells, Rd treatment resulted in increased mRNA expression of Smad2 in cultured 4T1 cells. Similarly, Rd treatment resulted in increased mRNA expression of Smad2 in 4T1-derived tumors compared to that detected in vehicle-treated mice (Fig. 7D). The expression of TGFβ1, TGFβR1, Smad3 and Smad4 was also examined. The mRNA level of TGFβ1 was decreased as a result of Rd treatment in cultured 4T1 cells but not in 4T1 cell-derived tumors. Rd treatment resulted in marginal increased in Smad3 mRNA expression in 4T1 cells but no significant changes in Smad3 expression was found in 4T1-derived tumors in Rd-treated mice. No significant changes were observed in the mRNA levels of TGFβR1 and Smad4 in either Rd-treated 4T1 cells or 4T1 cell-derived tumors from Rd-treated mice (see Supplementary Fig. S8). Moreover, the protein levels of TGF-β1, TGFβR1, Smad2, Smad3 and Smad4 were also examined. Rd treatment resulted in decreased protein level of TGFβ1 (Fig. 8A,B) and increased protein level of Smad2 in cultured 4T1 cells (Fig. 8A,D). Similar results were also observed for the protein levels of TGFβ1 (Fig. 9A,B) and Smad2 (Fig. 9A,D) in 4T1 cell-derived tumors. No significant changes in the protein levels of TGFβR1, Smad3 and Smad4 were observed either in cultured 4T1 cell (Fig. 8A,C,E,F) or 4T1 cell-derived tumors (Fig. 9A,C,E,F). These results consistently showed that Smad2 expression at both mRNA and protein levels is increased by Rd treatment in cultured 4T1 cells and in 4T1-derived tumors. In the meantime, Rd treatment also results in decreased protein level of TGFβ1 both *in vitro* and *in vivo*. To further address the implication of Smad2 in 4T1 cell migration, Smad2 was overexpressed in 4T1 cells (see Supplementary Fig. S9A) and migration kinetics was monitored for 24 h. The results showed that enhanced expression of Smad2 led to significant attenuation of migration in 4T1 cells (see Supplementary Fig. S9B,C).

### Rd treatment abrogated miR-18a-mediated suppression of Smad2 in 4T1 cells

**Figure 10:**
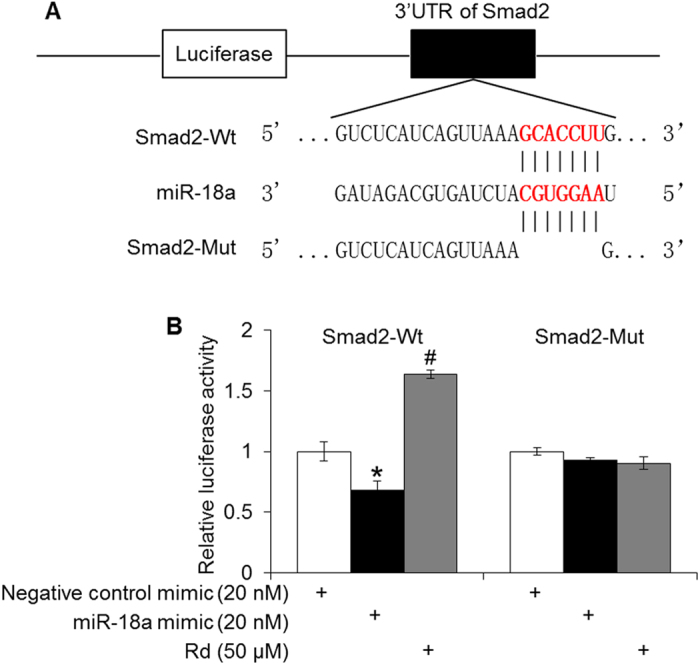
Rd derepressed miR-18a-mediated downregulation of Smad2 in 4T1 cells. (**A**) Scheme of pmirGLO luciferase reporter constructs of Smad2 3′-UTR containing wild type miR-18a target sites (Wt), deletion of seed region (Mut). The seed region is in bold and red. (**B**) Relative luciferase activity was quantified after transfection of miR-18a mimic or negative control mimic in the absence or presence of Rd. *Compared to that from the negative control mimic, *p* < 0.05; compared to that from the miR-18a mimic, *p* < 0.05.

Next, miR-18a-mediated regulation of Smad2 was verified in 4T1 cells. As shown in Fig. 10, miR-18a mimic transfection resulted in 32% reduction in the luciferase reporter activity of Smad2 wt 3′-UTR compared to that from the 4T1 cells transfected with negative control mimic. However, this miR-18a-mediated suppression of Smad2 3′-UTR luciferase reporter activity was not observed when the seed region of miR-18a was mutated. Furthermore, Rd treatment resulted in increased luciferase activity of Smad2 wt 3′-UTR but not that of Smad2 mut 3′-UTR in 4T1 cells. These results not only confirmed that Smad2 is a direct target of miR-18a in 4T1 cells, but also indicated miR-18a-mediated suppression of Smad2 expression is abrogated by Rd treatment.

## Discussion

The current study demonstrates that Rd treatment inhibits 4T1 cell migration and invasion *in vitro* and breast cancer lung metastasis in 4T1 cell-inoculated mice. Rd treatment also leads to decreased expression of miR-18a and increased mRNA and protein levels of Smad2 in both cultured 4T1 cells and 4T1 cell-derived tumors. Moreover, Smad2 is validated as a direct target of miR-18a and Rd treatment specifically abrogates miR-18a-mediated suppression of Smad2 in 4T1 cells.

TGFβ signaling is frequently altered in different types of tumor^32^. TGFβ1 has been shown to be overexpressed in human breast tumor and its expression level correlates with metastasis of breast cancer^33^. Smad2 and Smad3 play differential roles in executing TGFβ1 signaling resulting in either suppression or promotion of breast cancer progression. Smad2 knockdown increases the aggressiveness of metastatic human breast cancer MDA-MB-231 cells while Smad3 knockdown prolongs the latency and delays the growth of bone metastasis, indicating that selective targeting of Smad2 or Smad3 may result in different therapeutic responses^34^. It is thus worth noting that therapeutic strategies under development are mostly focused on abrogating the activities of TGFβ receptors, which may lead to complete blockade of TGFβ signaling mediated by both Smad2 and Smad3^35^. Our study demonstrates that in 4T1 cells and 4T1 cell-derived tumors, Rd treatment exhibits differential effects on the levels of TGFβ signaling molecules, e.g., Rd decreases the level of TGFβ1, increases Smad2 expression but causes little changes in the expression levels of TGFβR1, Smad3 and Smad4. These results imply selective effects of Rd treatment on the level of TGFβ signaling molecules in 4T1 mammary carcinoma cells.

miRNA-mediated gene expression regulation has been extensively studied and revealed to be important mechanism implicated in tumorigenesis and metastasis. Dysregulation of miRNAs is now considered as a common characteristic of nearly all human tumors^36^. miRNA machineries have been noted to function in a species or cell type specific and context-dependent manner, which necessitates the validation of miRNA/target interaction in the cell type under investigation. As aforementioned, miR-18a plays oncogenic roles in various types of cancer and Smad2 has been identified as a direct target of miR-18a in human neuroblastoma. Our results verify the direct interaction of miR-18a and Smad2, indicating that Smad2 is a gene target of miR-18a in 4T1 mouse mammary carcinoma cells. Rd treatment results in increased expression of Smad2, decreased expression of miR-18a and increased activity of the luciferase reporter of Smad2 3′-UTR containing intact miR-18a seed region. Together with the impact of Rd treatment on the expression of TGFβ signaling molecules, the anti-metastatic activity of Rd could implicate at least two mechanistic arms including derepressing miR-18a-mediated Smad2 regulation and lowering the expression of TGFβ1, with the latter remains to be further investigated for the involved mechanisms in the future studies.

Although Rd treatment resulted in significantly impaired cell viability in cultured 4T1 cells at higher doses (Fig. 1), it is noted the metastatic potential of 4T1 cells was susceptible to Rd treatment whether or not cell viability was factored in (Figs 2 and 3). Moreover, suppressed migration and invasion were observed in the absence of evident impairment in cell viability when 4T1 cells were exposed to Rd at 50 μM (Figs 1, 2 and 3). Moreover, Rd treatment led to significantly increased expression of anti-metastatic Timp2 in 4T1 cells at all the doses examined (see Supplemental Fig. S3).

PNS suppresses breast cancer metastasis. Our work here shows that Rd treatment alone is anti-metastatic during breast cancer progression, providing evidence supporting that Rd contributes to the pharmacological activity of PNS in attenuating breast cancer metastasis. We have previously shown that other naturally occurring chemical compound in PNS including ginsenoside Rb1, Rg1 and notoginsenoside R1 inhibits the growth of tumors derived from inoculated Lewis lung carcinoma cells when administered individually in the mice^22^. Another study has also indicated that ginsenoside Rg3 induces apoptosis in human breast cancer cells^37^. Future studies are required to evaluate the effects of these compounds in the model of breast cancer metastasis. It would also be interesting to study whether synergistic actions of Rd and other chemical compounds contained in PNS could be at work to reach greater suppression of breast cancer metastasis. Additionally, whether TGFβ1 level and miR-18a/Smad2 interaction are differentially impacted by these treatment modalities remains to be further investigated.

Our current study therefore demonstrates for the first time that Rd treatment attenuates metastatic features of breast cancer both in cultured 4T1 cell *in vitro* and in 4T1 cell-inoculated mice *in vivo*. This anti-metastatic pharmacological activity of Rd mechanistically in part implicates downregulated expression of TGFβ1 and derepression of miR-18a-regulated Smad2 expression in 4T1 cells. These results warrant further evaluation of Rd as a therapeutic agent in the management of breast cancer metastasis.

## Methods

### Cell culture

4T1 cell line and human breast cancer MDA-MB-231 cell line were purchased from the Chinese Academy of Sciences Cell Bank of Type Culture Collection (Shanghai, China). 4T1 cells were cultured in RPMI-1640 medium supplemented with 10% FBS (Gibco, USA), 100 U/ml penicillin and 100 μg/ml streptomycin (Life Sciences, USA). MDA-MB-231 cells were cultured in DMEM medium supplemented with 10% FBS (Gibco, USA), 100 U/ml penicillin and 100 μg/ml streptomycin (Life Sciences, USA). Cells were maintained in a humidified incubator at 37 °C in a 5% CO_2_ atmosphere.

### Cell viability assay

4T1 cells were seeded in triplicate culture at the number of 1 × 10^4^ cells per well in a 96-well plate with vehicle or Rd (purity >98%, Shanghai Yuanye Biotechnology Co., Ltd, China) at indicated concentrations. To assess the cell viability, the percentage confluence was monitored at 1 h interval using the high definition automated imaging system from IncuCyte following the manufacturer’s direction (Essen Bioscience, USA)^38^. Human breast cancer MDA-MB-231 cells were seeded in a 96-well plate at the number of 1 × 10^4^ cells per well. Cells were treated with vehicle or Rd at indicated concentrations. Cell viability was examined 72 h later by methyl thiazolyl tetrazolium (MTT) (Sigma-Aldrich, USA) assay. Briefly, at the end of experiment, cells were incubated with MTT at 0.5 mg/mL for 4 h and the absorption value of formazan crystal was analyzed at 490 nm using a microplate reader (Bio-Tek, USA).

### Cell migration assay

Cell migration was evaluated by a scratch-wound assay using IncuCyte (Essen Bioscience, USA) as previously described. Briefly, 4T1 cells were plated into Essen ImageLock 96-well plates at the number of 2 × 10^4^ cells per well. Cells were then serum-starved for six hours and the monolayer of confluent cells was scratched using Essen Wound Maker to generate wound approximately 600 μm wide. After wounding, debris was removed by PBS washing for two times, which was followed by addition of vehicle or Rd at indicated concentrations. Typical kinetic updates were recorded at 1 h intervals for the duration of 48 h. To assess the impact of miR-18a expression on 4T1 cell migration, 4T1 cells were seeded in Essen ImageLock 96-well plates at the number of 2 × 10^4^ cells per well. Cells were then transfected with negative control mimic or miR-18a mimic at concentration of 50 nM for 24 h. The monolayer of confluent cells was then scratched using the Essen WoundMaker to generate wound approximately 600 μm wide. After wounding, debris was removed by PBS washing for two times. Wound images were automatically acquired by the IncuCyte Imaging System using time-lapse bright field microscopy. Typical kinetic updates were recorded at 2 h intervals for the duration of 12 h. To examine the 4T1 cell migration in the context of enhanced expression of Smad2, 4T1 cells were plated into Essen ImageLock 96-well plates at 2 × 10^4^ cells per well, which was followed by transfection of Smad2/pCDNA3.1 or pCDNA3.1 vector control (100 ng per well). The monolayer of confluent cells was then scratched using Essen WoundMaker to generate wound approximately 600 μm in width. Typical kinetic updates of wound images were recorded by the IncuCyte ZOOM imaging system using time-lapse bright field microscopy at the interval of 1 h for 24 h. The data were analyzed using an integrated metric of Incucyte, relative wound density (RWD). This algorithm measures cell density in the wound area relative to the cell density outside of the wound area at each set time point. At t = 0, the RWD is set to be zero. When the cell density inside of the wound is the same as the cell density outside of the initial wound, the RWD is 100%.

### Cell invasion assay

Cell invasion assay was performed as previously described^39^ with slight modifications. Briefly, triplicate cultures of 4T1 cells in the number of 1 × 10^5^ were resuspended in 100 μl of serum-free RPMI-1640 medium containing vehicle or Rd at indicated concentrations. The resuspended cells were seeded in the top chamber of the transwell with or without coating by 30 μl of serum-free RPMI 1640 medium-diluted matrigel (BD Biosciences, USA). RPMI-1640 culture medium supplemented with 10% FBS was added to the lower chamber to serve as chemoattractant. Cell migration was assessed in the transwells without matrigel coating while invasion was evaluated in the presence of matrigel coating. After incubation at 37 °C for 24 h, viable cells were first determined using the CellTiter 96 AQueous Non-Radioactive Cell Proliferation MTS assay (Promega, USA) following the manufacturer’s instructions. Cells migrated or invaded through the filters were assessed by staining using 0.5% crystal violet solution, which was followed by absorbance measurement at 540 nm using a microplate reader (Bio-Teck, USA). Cell migration was assessed by normalizing against proliferation. Cell invasion was evaluated by normalizing against migration. A series of pictures for each insert were also captured, and merged to a whole well image under light microscope (Leica, Germany). Cell invasion assay was also performed and recorded by Incucyte. Briefly, 4T1 cells were seeded in Essen ImageLock 96-well plates at the number of 2 × 10^4^ cells per well. Cells were then transfected with negative control mimic or miR-18a mimic at concentration of 50 nM for 24 h. The monolayer of confluent cells was then scratched using the Essen WoundMaker to generate wound approximately 600 μm wide and 50 μl of serum-free RPMI 1640-diluted matrigel was coated on the wound. Wound images were automatically acquired by the IncuCyte Imaging System using time-lapse bright field microscopy. Typical kinetic updates were recorded at 2 h intervals for the duration of 24 h. The data were then analyzed using an integrated metric of Incucyte RWD.

### Cell cycle analysis

After indicated treatment, 4T1 cells were collected and resuspended in 0.4 ml PBS, which was followed by addition of 0.7 ml absolute alcohol containing 3% serum for fixation at 4 °C overnight. RNase-A was added to a final concentration of 50 μg/ml and digestion was performed in a water bath for 30 min at 37 °C. Propidium iodide (PI, BD Biosciences, USA) was added at a final concentration of 10 μg/ml and the staining was carried out in dark in an ice bath for 30 min. After resuspension of cells, the cell cycle distribution was acquired and the percentage of cells in each cell cycle phase was quantified by BD FACSVerse flow cytometer (BD Biosciences, USA).

### Animals

Female BALB/c mice (6–8 weeks) were obtained from the Sino-British SIPPR/BK Laboratory Animal Ltd (Shanghai, China; Certificate. No. SCXK Shanghai 2008–0016). Mice were housed in a standard polypropylene cage containing sterile bedding under a controlled condition of temperature, humidity, and light (12 h light/dark cycle) in the laboratory animal center in Yueyang Hospitial, Shanghai University of TCM (No. SYXK [Hu] 2011–0109). All animal handling and experimental procedures were approved by the Shanghai University of TCM Institutional Animal Care and Use Committees. The methods were carried out in accordance with the approved guidelines and regulations.

### Tumor growth assay

Tumor implantation model was established as previously described with slight modifications. Briefly, 4T1 cells in the number of 1 × 10^6^ cells were resuspended in 0.1 ml PBS and injected into the inguinal mammary fat pad of each mouse. BALB/c mice were then randomly divided to receive Rd or vehicle treatment. Each mouse received either daily Rd treatment at the dose of 50 mg/kg bw or 0.9% saline treatment through intraperitoneal injection, which was followed by measurements of body weight and tumor size. Tumor size was measured every other day in two perpendicular dimensions (L = length, W = width) with a vernier caliper and recorded as volume (mm^3^) calculated by L × W^2^/2.

### Spontaneous tumor metastasis assay

Spontaneous tumor metastasis model was established as previously described with slight modifications. Briefly, 4T1 cells resuspended in 0.1 ml PBS were injected into the inguinal mammary fat pad of BALB/c mice at the number of 1 × 10^6^ cells per mouse. Mice were then randomly divided to different groups and treated daily with either Rd at the dose of 50 mg/kg bw or 0.9% saline by intraperitoneal injection. The experiment was terminated 3 weeks later and the number of lung metastasis nodules was counted. Primary tumors were dissected and subjected to expression analyses in subsequent experiments.

### Experimental tumor metastasis assay

The experimental metastasis model was established as previously described. In brief, 4T1 cells in the number of 5 × 10^5^ cells were resuspended in 0.1 ml PBS and delivered to each female BALB/c mouse via lateral tail vein injection. Mice were then randomly divided to different groups and treated daily with either Rd at the dose of 50 mg/kg bw or 0.9% saline by intraperitoneal injection. Body weight and survival rate were measured and recorded. The experiment was terminated 2 weeks later and the number of lung metastasis nodules was counted.

### Histology examination

After euthanizing the mice, the lungs were removed and perfused with buffered formalin through cannulated trachea and further processed for histological examination. Paraffin-embedded luntg sections 4 μm hick were stained with hematoxylin and eosin (H&E). Tumor lesions in the lung were quantified by measuring the total tissue area per lung section (D1) and the number of micro-metastasis present in the same area (D2) using Image-Pro Plus software. The metastatic index was calculated by the ratio of D2/D1^40^. The number of lung colonies was counted by light microscopy at the magnification of 50× (Leica, Germany).

### Real-time PCR

Total RNA from 4T1 cells or homogenized tumor samples was extracted using miRNeasy Mini Kit (Qiagen, Germany) followed by reverse transcription using miScript RT II Kit (Qiagen, Germany) according to the manufacturer’s instructions. Relative expression of candidate genes or miRNAs was analyzed using Roche SYBR Green PCR Kit on the LightCycler 480 (Roche). The 2^−ΔΔCt^ method^41^ was used for data analysis, with miRNA levels normalized to RNU6B and mRNA levels normalized to GAPDH, respectively. For miRNA expression analyses, the miRNA-specific forward primers and the universal reverse primers were used. The miR-18a-specific forward primer sequences were 5′-TAAGGTGCATCTAGTGCAGATAG-3′, and the forward primer sequences of reference RNU6B were 5′-ACGCAAATTCGTGAAGCGTT-3′. The primer sequences for gene expression analyses included: Timp2 forward primer: 5′-CTAATTGCAGGAAAGGCAGAAGG-3′ and reverse primer: 5′-AAGGGATCATGGGACAGCGA-3′; MMP3 forward primer: 5′-GACTCAAGGGTGGATGCTGT-3′ and reverse primer: 5′-GGATGCCTTCCTTGGATCTCT-3′; TGFβ1 forward primer: 5′-AGCTGCGCTTGCAGAGATTA-3′ and reverse primer: 5′-AGCCCTGTATT CCGTCTCCT-3′; TGFβR1 forward primer: 5′-TGAATCCTTCAAACGCGCTG-3′ and reverse primer: 5′-TGGCCTTAACTTCTGTTCGCA-3′; Smad2: forward primer 5′-TGTAGACGGCTTCACAGACC-3′ and reverse primer: 5′-TCTGGTTACAGTTGGG GCTC-3′; Smad3: forward primer 5′-CAGTCTCCCAACTGCAACCA-3′ and reverse primer: 5′-TGGTAGACAGCCTCAAAGCC-3′; Smad4 forward primer: 5′-CTTCAAGCTGCCCTGTTGTG-3′ and reverse primer: 5′-CACTAAGGCACCTGACCCAA-3′; and GAPDH forward primer: 5′-CCGGTGCTGAGTATGTCGTG-3′ and reverse primer: 5′-CCTTTTGGCTCCACCCTTC-3′.

### Western blot

4T1 cells or tumor samples were lysed in TNEN buffer containing proteinase inhibitor cocktail. Proteins were then denatured at 95 °C and loaded onto 12% SDS-PAGE gel, and transferred to PVDF membrane. After incubation with anti-β-Actin antibody (nb100-74340, Novus), anti-TGF-β1 antibody (Abcam, USA), anti-TGFβR1 antibody, anti-Smad2 antibody, anti-Smad3 antibody, or anti-Smad4 antibody (Cell Signaling, USA) at 4 °C overnight, the blots were incubated with HRP-conjugated secondary antibody (1:5000). Signals were visualized using ECL substrates (PerkinElmer, USA) and recorded by UVP BioSpectrum imaging system. The semi-quantitative analyses of the protein levels were performed by Launch VisionWorks LS. β-Actin was used as an internal control for normalization purpose.

### Luciferase reporter assay

A fragment of 3′-UTR of Smad2 in the length of 303 bp containing the putative miR-18a binding site was amplified by PCR from genomic DNA, and then subcloned at the SacI and XhoI sites into pmirGLO vector (Promega, USA) immediately downstream to the luciferase gene sequence. A mutant construct containing 3′-UTR of Smad2 with deletion of miR-18a seed region was also synthesized through overlap extension PCR (OE-PCR)^42^. PCR primer sequences for constructs were as following: Smad2 wild type (wt) forward primer: 5′-CGAGCTCGACCCATCAAAGACT CGCTGTA-3′ and reverse primer: 5′-CCGCTCGAGTGGGTCTGGACACATTACCTG-3′; SMAD2 mutation (mut) forward primer: 5′-CAGATTCCACTTTAACTGATGAGACCTCAA-3′ and reverse primer: 5′-ATCAGTTAAAGTGGAATCTGTTTCCTATAT-3′. The constructs were also confirmed by sequencing.

For luciferase activity assay, 4T1 cells in the number of 2 × 10^5^ were seeded in 24-well plates in DMEM supplemented with 10% FBS 12 h prior to transfection. Cells were transfected with 100 ng of reporter plasmid pmirGLO-Smad2 wt 3′-UTR or pmirGLO-Smad2 mut 3′UTR and 20 nM mmu-miR-18a mimic or negative control mimic in the presence or absence of 50 μM Rd. Cells were harvested 48 h later and the luciferase activity was analyzed using Dual-Luciferase reporter system following the manufacturer’s instructions (Promega). Luminescent signal was measured by luminometer (GloMax20/20, Promega) and each value from the firefly luciferase was normalized by that of the Renilla luciferase.

### Statistical analysis

The data obtained from at least three independent experiments were presented as means ± S.E.M. Comparisons between two groups were performed using Student t-test with *p* value less than 0.05 being considered as statistically significant.

## Additional Information

**How to cite this article**: Wang, P. *et al*. Ginsenoside Rd attenuates breast cancer metastasis implicating derepressing microRNA-18a-regulated Smad2 expression. *Sci. Rep.* **6**, 33709; doi: 10.1038/srep33709 (2016).
